# The Impact of Non-Lethal Single-Dose Radiation on Tumor Invasion and Cytoskeletal Properties

**DOI:** 10.3390/ijms18092001

**Published:** 2017-09-18

**Authors:** Tim Hohmann, Urszula Grabiec, Carolin Vogel, Chalid Ghadban, Stephan Ensminger, Matthias Bache, Dirk Vordermark, Faramarz Dehghani

**Affiliations:** 1 Institute of Anatomy and Cell Biology, Martin Luther University Halle-Wittenberg, Grosse Steinstrasse 52, 06108 Halle, Germany; tim.hohmann@medizin.uni-halle.de (T.H.); urszula.grabiec@medizin.uni-halle.de (U.G.); carolin.vogel@student.uni-halle.de (C.V.); chalid.ghadban@medizin.uni-halle.de (C.G.); 2 Department of Radiation Oncology, Martin Luther University Halle-Wittenberg, Ernst-Grube-Strasse 40, 06120 Halle, Germany; stephan.ensminger@uk-halle.de (S.E.); matthias.bache@uk-halle.de (M.B.); dirk.vordermark@uk-halle.de (D.V.)

**Keywords:** glioblastoma, cell mechanics, slice cultures, actin, phalloidin, radiation, cytoskeleton

## Abstract

Irradiation is the standard therapy for glioblastoma multiforme. Glioblastoma are highly resistant to radiotherapy and the underlying mechanisms remain unclear. To better understand the biological effects of irradiation on glioblastoma cells, we tested whether nonlethal irradiation influences the invasiveness, cell stiffness, and actin cytoskeleton properties. Two different glioblastoma cell lines were irradiated with 2 Gy and changes in mechanical and migratory properties and alterations in the actin structure were measured. The invasiveness of cell lines was determined using a co-culture model with organotypic hippocampal slice cultures. Irradiation led to changes in motility and a less invasive phenotype in both investigated cell lines that were associated with an increase in a ”generalized stiffness” and changes in the actin structure. In this study we demonstrate that irradiation can induce changes in the actin cytoskeleton and motility, which probably results in reduced invasiveness of glioblastoma cell lines. Furthermore, “generalized stiffness” was shown to be a profound marker of the invasiveness of a tumor cell population in our model.

## 1. Introduction

Malignant tumors cause millions of death per year, and the trend is increasing [1,2]. Among all known tumor types, glioblastoma multiforme (GBM) is one of the most aggressive regarding proliferation, infiltration, and survival. Even though GBM are highly resistant to radiotherapy, this treatment is still considered standard [3,4,5,6,7,8]. A growing set of evidence exists for the effects of radiation on a cellular level that are often associated with the invasive properties of tumor cells. The invasion of tumor cells into the tissue is embedded in a multitude of processes demanding enormous structural changes of a tumor cell. This includes an initial reduction in cell adhesion, a degradation of the surrounding extracellular matrix, and a subsequent (directed) movement away from the main tumor. 

One structure highly involved in the abovementioned processes, and thus in tumor invasion, is the cytoskeleton. Closely related to the cytoarchitecture are mechanical properties [9,10], differing for tumor cells compared to non-tumor cells [11,12,13,14]. Furthermore, for tumor cells it was demonstrated that the mechanical alterations are necessary for tumor progression as well [15,16,17].

Irradiation of GBM cell lines can lead to a deregulation of genes associated with the organization of the cytoskeleton, as well as cell–cell and cell–matrix adhesion [18,19,20]. A study on two cytoskeletal components, namely β-actin and α-tubulin, in T98G and U87MG glioblastoma cells revealed no alterations after irradiation (8 Gy) at mRNA and protein level after 30 min and 6 h [19]. In contrast, the human dermal microvascular endothelial cell line DMEC, but not the human umbilical vein endothelial cell line HUVEC, responded with actin cytoskeletal reorganization after irradiation (0.5–20 Gy) after 10 min, and the rearrangements persisted after 24 h [21]. Another study demonstrated an increased expression of the adhesion molecules β1- and β3-integrin in a dose-dependent manner in the GBM cell lines A172 and U138, but not in LN229 and LN18 after doses of 2 or 6 Gy [22]. In contrast, a further study reported on a negative role of irradiation on the maturation of focal adhesions in C6 rat glioma cells [23]. These findings hint at a cell line or tumor specific impact of irradiation on the organization of the cytoskeleton and cell adhesion. An actual analysis of the adhesive properties or a quantitative analysis of the actin cytoskeleton structure was not performed. 

In this work we investigate the effect of single-dose radiation of 2 Gy on cell properties related to cytoskeletal dynamics, such as the cell motility and cell stiffness, but also directly on the structure of the actin cytoskeleton. Furthermore, a relationship was established between the abovementioned parameters and the invasive properties of GBM cell lines using a co-culture model. Additionally, the effect of single-dose irradiation on cell proliferation and survival has been studied. 

## 2. Results

### 2.1. Impact of Radiation on Cell Proliferation and Cell Death

**Figure 1:**
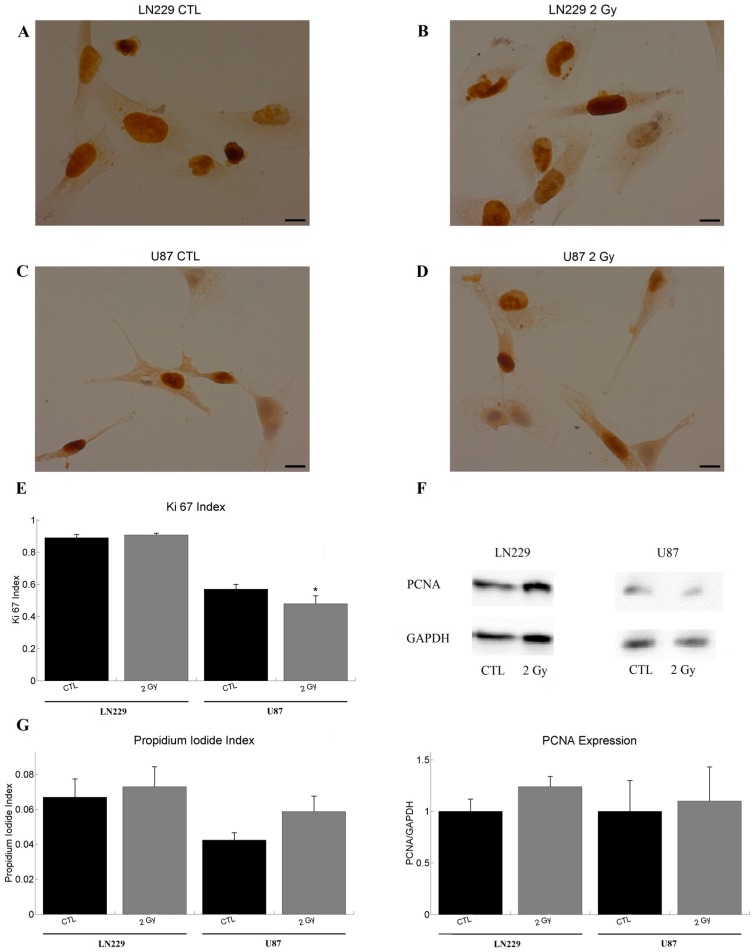
Influence of irradiation on cell division and death. (**A**,**B**) representative images of the Ki67 staining of LN229 cells with and without irradiation with 2 Gy. Most of the cells were found to be proliferating; (**C**,**D**) depicts a representative sample of the Ki67 staining of U87 cells with and without irradiation with 2 Gy. One can observe a slightly decreased number of Ki67 positive cells compared to the control group. LN229 (**A**,**B**) cells are more proliferative than U87 (**C**,**D**) cells; (**E**) illustrates the respective Ki67 index (Ki67 positive cells/cell number) of LN229 (sample size: *n*_CTL_ = 20; *n*_2Gy_ = 16) and U87 (*n*_CTL_ = 21; *n*_2Gy_ = 27) cells before and after irradiation. Irradiation decreased the proliferation rate of U87 only; (**F**) shows the PCNA expression of LN229 (*n*_CTL_ = 5; *n*_2Gy_ = 5) and U87 (*n*_CTL_ = 4; *n*_2Gy_ = 4) cells, normalized to the control groups and a representative blot with GAPDH at 37 kDa and PCNA at 36 kDa. No significant effects were observed; (**G**) visualizes the ratio of propidium iodide positive (dead) cells to the total number of cells for LN229 (*n*_CTL_ = 10; *n*_2Gy_ = 10) and U87 (*n*_CTL_ = 10; *n*_2Gy_ = 10). No significant effect was observed. The graphs show the mean value together with the standard error of the mean (sem). Statistics was performed using *t*-test and significance was chosen for *p* < 0.05. The asterisk denotes significant results regarding the control measurement of the same cell line. Scale bar corresponds to 10 µm.

The Ki67 staining of LN229 cells showed a similar proliferation status before and after irradiation (Figure 1A,B,E). The amount of proliferating U87 cells was lower compared to that of LN229 cells (Figure 1A–E). In contrast to LN229 cells (Ki67_CTL_ = 0.89; Ki67_2Gy_ = 0.91), the proliferation rate of U87 cells decreased significantly after irradiation (Ki67_CTL_ = 0.57; Ki67_2Gy_ = 0.48; Figure 1C–E). The Western blot revealed that the proliferating cell nuclear antigen (PCNA) amount was not significantly altered by the irradiation neither for LN229 (PCNA_CTL_ = 1; PCNA_2Gy_ = 1.24) nor for U87 (PCNA_CTL_ = 1; PCNA_2Gy_ = 1.1) cells (Figure 1F). The propidium iodide (PI) staining revealed no change in the ratio of dead cells before and after irradiation for both LN229 (PI_CTL_ = 0.07; PI_2Gy_ = 0.07) and U87 (PI_CTL_ = 0.04; PI_2Gy_ = 0.06) cells (Figure 1G).

### 2.2. Analysis of Mechanical Properties by Atomic Force Microscopy

**Figure 2:**
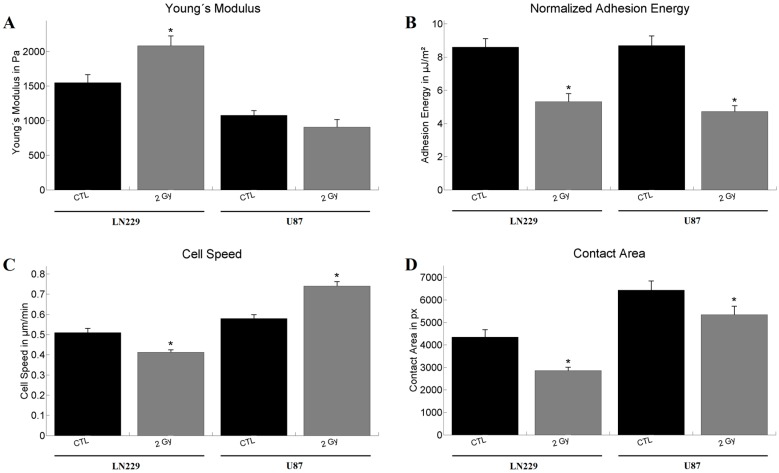
Results of the atomic force microscopy (AFM) and live cell imaging measurements for LN229 and U87 (**A**) Shows the Young’s modulus. Statistical significance was found after irradiation of LN229 cells (*n*_CTL_ = 60; *n*_2Gy_ = 35) only. U87 (*n*_CTL_ = 60; *n*_2Gy_ = 35) cells did not show a change in elasticity; (**B**) Calculated mean adhesion energies using the Derjarguin-Muller-Topolov model. Irradiation led to a decrease in adhesion for both cell lines. The number of analyzed cells used for determination of adhesion energy were identical to those for the Young’s modulus; (**C**) Derived speeds from the time laps images. Inverse effects could be observed for the two cell lines. LN229 reacted with a decrease (*n*_CTL_ = 118; *n*_2Gy_ = 247), while irradiation of U87 led to an increase in cell speed (*n*_CTL_ = 158; *n*_2Gy_ = 118); (**D**) Regarding the contact area of the cells after irradiation both cell lines reacted with a decrease in area. The numbers of experimental values for the cell area were identical to the ones for the cell speed. The graphs show the mean value together with the standard error of the mean. Statistical analysis was performed using the Mann–Whitney test and significance was chosen for *p* < 0.05. The asterisk denotes significant results regarding the control measurement of the same cell line.

For the atomic force microscopy (AFM) measurements weakly adherent cells were used, approximately 15 min after seeding. Regarding the Young’s modulus *E*, the irradiation had an effect on LN229 cells only, leading to an increase in the modulus (*E*_CTL_ = 1584 Pa; *E*_2Gy_ = 2083 Pa). In U87 cell no significant change in the Young’s modulus was observed (*E*_CTL_ = 1079 Pa; *E*_2Gy_ = 906 Pa; Figure 2A). The measured adhesion energies *U*, defined by the needed energy to detach the cantilever from the cell surface, were decreased for both cell types after irradiation with 2 Gy (Figure 2B). For LN229 cells the adhesion energy was reduced from *U*_CTL_ = 8.6 µJ/m^2^ to *U*_2Gy_ = 5.3 µJ/m^2^ and for U87 from *U*_CTL_ = 8.7 µJ/m^2^ to *U*_2Gy_ = 4.7 µJ/m^2^.

### 2.3. Analysis of Motile Properties Using Time Lapse Imaging

The live cell experiments showed a decrease in speed *v* for LN229 after irradiation from *v*_CTL_ = 0.51 µm/min to *v*_2Gy_ = 0.41 µm/min, while an increase was measured for U87 cells (*v*_CTL_ = 0.58 µm/min; *v*_2Gy_ = 0.74 µm/min; Figure 2C). The analysis of the contact area *A* for each cell type and treatment revealed for both cell lines a reduced contact area after the irradiation. In case of LN229 cells the area decreased from *A*_CTL_ = 4354 px to *A*_2Gy_ = 2861 px and for U87 from *A*_CTL_ = 6433 px to *A*_2Gy_ = 5348 px (Figure 2D).

### 2.4. Tumor Invasion Measurements

**Figure 3:**
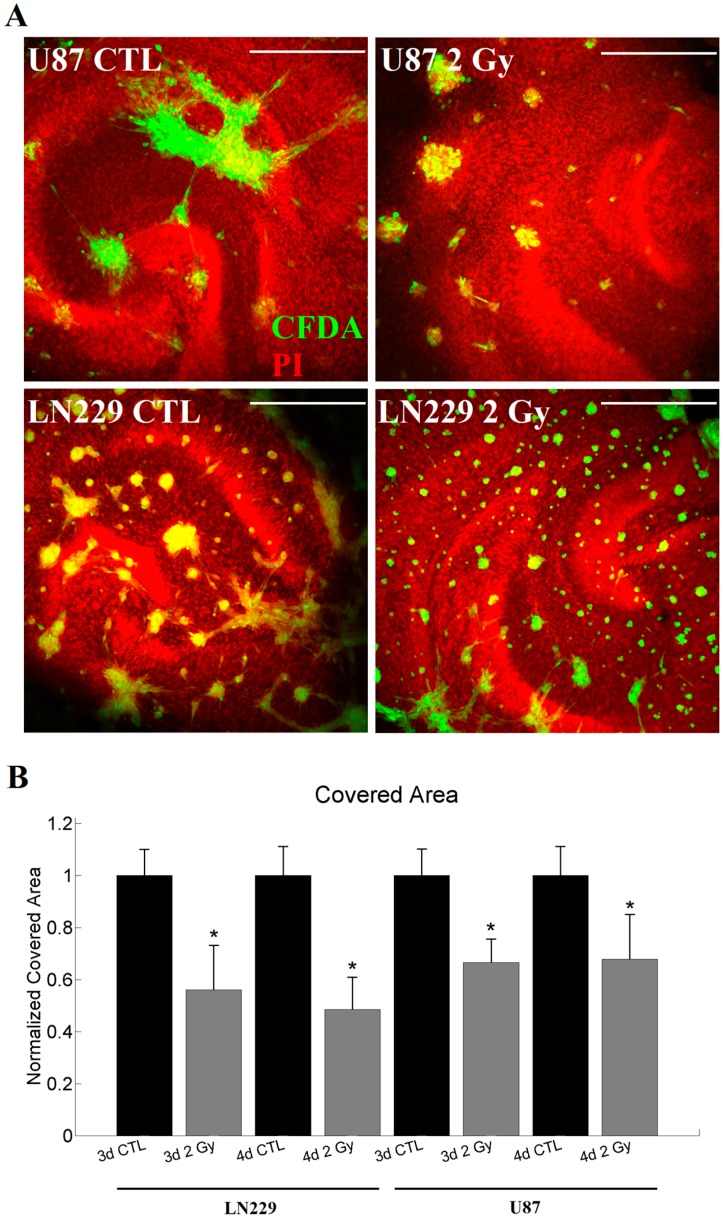
Measurements of the invasion for LN229 and U87. (**A**) represents a typical invasion pattern generated by LN229 and U87 with or without irradiation after four days of invasion. In red the propidium iodide dyed cytoarchitecture of the OHSC is visualized (labeled PI), while green depicts the tumor labeled using carboxyfluorescin diacetate (labeled CFDA); (**B**) For both, LN229 (*n*_3dCTL_ = 37; *n*_3d2Gy_ = 22; *n*_4dCTL_ = 41; *n*_4d2Gy_ = 20) and U87 (*n*_3dCTL_ = 53; *n*_3d2Gy_ = 30; *n*_4dCTL_ = 51; *n*_4d2Gy_ = 16) cells, and 3 or 4d invasion time the irradiation led to a significant decrease in the invasiveness. Statistics was performed using the Mann–Whitney test and significance was chosen for *p* < 0.05. The asterisk denotes significant results regarding the control measurement of the same cell line. The scale bar corresponds to 400 µm.

Both tested cell lines showed invasive behavior in organotypic hippocampal slice cultures (OHSC) and formed tumors. The tumor cells were scattered over the slice cultures sometimes forming network-like structures (Figure 3A). The invasiveness *A* of each tumor was determined by the area covered by tumor cells in relation to the area of OHSC, normalized to the respective control measurement. Here again, both cell lines reacted qualitatively in the same way. For both, LN229 cells (*A*_3d2Gy_ = 0.56; *A*_4d2Gy_ = 0.48) and U87 cells (*A*_3d2Gy_ = 0.67; *A*_4d2Gy_ = 0.68), a significant decrease in invasion was observed after the irradiation for three and four days of invasion (Figure 3B).

### 2.5. Network Analysis of Single Cell Properties and Composite Parameters

**Figure 4:**
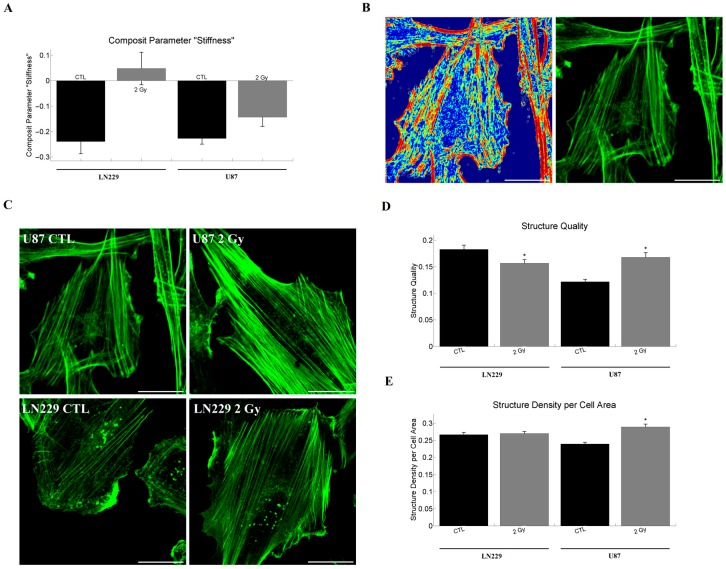
Barplot of the composite parameter “stiffness” and actin structure measurements. (**A**) The composite parameter “stiffness” is in both cases strongly increased after irradiation; (**B**) depicts the structure image as a heat map on the left and the respective phalloidin staining on the right for U87 control cells. A correspondence of highly structured regions in the actin staining with the respective structure image is visible. Furthermore, unstructured, homogeneous regions do not contribute to the structure image, as for example in the center of the image.; (**C**) displays sample images of actin staining for U87 and LN229 cells with and without irradiation. A dense actin network is visible for both cell lines and the respective treatments; (**D**) shows the quantification of the quality of actin structures. A decrease after irradiation is found in the case of LN229 (n_CTL_ = 52; n_2Gy_ = 66) cells, while an increase is observed for U87 (n_CTL_ = 124; n_2Gy_ = 94) cells; (**E**) illustrates the changes of actin structure density after irradiation. Only in U87 cells an increase in structure density could be observed. The sample size is identical to the one of the quality measurements. Statistics was performed using the Mann–Whitney test and significance was chosen for *p* < 0.05. The asterisk denotes significant results regarding the control measurement of the same cell line. The scaling corresponds to 30 µm.

The obtained parameter groupings were the same as published before and are given in the Figure S1, Tables S1 and S2 [24]. Most notably, the Young’s modulus and the indentation depth formed a cluster. Both cell lines responded with an increase of the dimensionless composite parameter formed by indentation and Young’s modulus (called: composite parameter “stiffness”; *S*). In the case of LN229 cells the increase was from *S*_CTL_ = −0.24 to *S*_2Gy_ = 0.05 and for U87 from *S*_CTL_ = −0.23 to *S*_2Gy_ = −0.14, respectively (Figure 4A).

### 2.6. Analysis of Actin Cytoskeleton Organization in Adherent Cells

The analysis of the actin staining revealed the expected structure and dense actin network of the glioblastoma cells (Figure 4B,C). We observed a clearly visible peripheral actin structure and dense arrays of mostly parallel stress fibers. Protrusive actin appeared as dense clusters at cell edges, while punctuate actin appeared as bright dots inside the cytoplasm. For LN229 cells we could observe a decrease in the quality *q* of structure (*q*_CTL_ = 0.183, *q*_2Gy_ = 0.157, Figure 4D), but no effect on the structure density *ρ* (*ρ*_CTL_ = 0.267, *ρ*_2Gy_ = 0.270, Figure 4E) after irradiation. In contrast, the irradiation of U87 cells led to an increase in structure quality (*q*_CTL_ = 0.122, *q*_2Gy_ = 0.168, Figure 4D) and density (*ρ*_CTL_ = 0.239, *ρ*_2Gy_ = 0.289, Figure 4E).

## 3. Discussion

### 3.1. Effects of 2 Gy Single-Dose Radiation on Cell Proliferation and Survival

In this study we examined the effect of a single 2 Gy irradiation event on cell proliferation and survival. A reduced proliferation was detected for U87 cells only. Since LN229 express mutant p53 and U87 cells express wild-type p53 [25] it might be one possible explanation for the different behavior after irradiation, as p53 is discussed as a factor modulating radiosensitivity [26,27,28,29,30,31]. Recently, a G2 arrest was observed for LN229 and U87 cells after irradiation, but only for doses of 5 Gy or higher [32]. In accordance to our data, a 2.18 Gy irradiation of U87 led to a reduced growth rate that resolved partly over time [33]. For LN229 cells, effects on cell cycle and growth rate after 2.18 Gy irradiation were absent after approximately 20 to 40 h [33]. As the time point of measurement in our experiments was 48 h after irradiation, our data on proliferation are in agreement with those obtained by Combs et al. [33]. Furthermore, we could not detect a significant effect on cell survival for both cell lines, which is in agreement with previously reported results by other groups, showing that significantly higher radiation doses are necessary to induce apoptosis [28,34,35].

As glioma stem cells are considered to be more radio-resistant [7], it is expected that irradiation is favoring their survival and proliferation. Both cell lines used have a different expression profile of cancer stem cell markers and do not express all of them [36,37]. This is of special importance because the study of Bao et al. demonstrated that irradiation seems to enrich glioma stem cells, but not to generate them de novo [7]. Given the negligible death rate and comparably low radiation dose used, it is unlikely that the fraction of glioma stem cells increased significantly during the short subsequent culture time.

Consequently, the observed effects of irradiation on invasiveness are not mediated via cell death and, in the case of LN229 cells, also not via proliferation.

### 3.2. Effects of 2 Gy Single-Dose Radiation on Single Cell Properties

The live cell measurements revealed an opposing effect for the two cell lines in terms of cell speed. While LN229 cells showed a reduction of the cell speed after irradiation, U87 cells moved faster compared to their non-irradiated state. Studies from other groups revealed that irradiation can lead to an increased motility of glioma cells and it was argued that this effect is related to the activation of the small GTPases Rac1 and subsequent inhibition of RhoA [38,39]. Both RhoA and Rac1 are associated with rearrangements in the actin cytoskeleton, like stress fiber and adhesion formation or the generation of lamellipodial protrusions, and thus are coupled to the cells’ motility [40,41,42,43]. Since a 2 Gy photon beam irradiation led to epidermal growth factor receptor (EGFR) activation in U87 cells, which in turn activates Rac1 signaling, it is highly likely that the same mechanism is responsible for the increased motility of U87 after irradiation observed here [44,45,46,47]. This hypothesis is supported by our analysis of the actin structure, showing a higher actin structure density and quality in U87 cells. In contrast, LN229 cells did not show a change in EGFR activation after radiation and thus a different mechanism might be responsible for the decrease in cell speed [47]. In the case of LN229 cells, the increased elastic modulus might give an explanation for the decreased speed. If the elastic modulus of a cell rises, the force necessary to form protrusive structures for cellular movement increases as well and may thus impair motility [48]. Consequently, it is very likely that small GTPases are involved in the observed effects. However, alterations in the activity or amount of components of focal adhesions or actin-related proteins will possibly result in changes of the other system. For example, forces generated by actin polymerization and myosin-dependent contractility affect mechanosensitive proteins (e.g., talin, vinculin), integrins, actin-polymerizing elements (e.g., zyxin, formins), and other molecules such as FAK [49]. As we have observed changes in the actin structure and motility, there are several further possible molecular targets that might be responsible for the observed effects. 

The evaluation of the contact area revealed a decrease for both cell lines after irradiation. This might be a hint that both cell lines reacted with a loss of adhesion towards the substrate. In different glioblastoma cell lines, irradiation was found to deregulate up to 100 genes that were associated with cytoskeletal organization, tumor invasiveness, or adhesiveness [18,19,20]. While one study demonstrated an increase in β1- and β3-integrin in a dose-dependent manner [22], another investigation observed less matured focal adhesions after 12 Gy irradiation [23]. Other groups reported on missing significant effects on α4-integrin, E-cadherin, β-actin, and α-tubulin mRNA and protein levels after the irradiation of U251 glioblastoma cells [20]. These diverse results related to adhesion indicate the strong cell line dependence of irradiation effects. However, it has to be considered that the area in contact with the substrate is influenced by other factors like the cell volume, the actin–cortex tension, or the amount of contractile structures. The analysis of the structure of actin revealed a higher density of actin structures inside the U87 cells, hinting at a higher amount of stress fibers. Stress fibers are the most prominent structures observed in U87 and thus it points to increased contractility with a subsequently lower contact area. For LN229 cells, a similar argument as for the cell speed can be assumed, with a higher elastic modulus resulting in an increased force necessary to spread out the cell, thus leading to a lower contact area. 

In contrast to live cell measurements, the AFM analysis allowed a direct measurement of adhesion energies on a time scale of approximately 1 s. For both cell lines, the adhesion energy decreased after irradiation, being in agreement with previous results for the contact area. Given the time scale of approximately 1 s of our measurements and the cantilever material silicon nitride, it is unlikely that specific adhesion bonds were forming. Thus, an alteration of unspecific adhesion events might be responsible for the drop in cell–cantilever adhesion. Furthermore, the AFM measurements showed an increased Young’s modulus for LN229 cells after irradiation, while no effect was observed for U87 cells. To the authors’ knowledge, this is the first time the effect of irradiation on glioblastoma cells elastic modulus has been addressed. Studies in fibroblasts and transformed fibroblasts revealed no change in cellular stiffness 24 h after irradiation with 2 Gy for the fibroblast line BALBc/3T3 and an increase in SVT2 transformed BALBc/3T3 cells [50]. Another group found a decrease in cellular stiffness after carbon ion irradiation with 2 Gy in all but one of the hepatoma cell lines used [51]. Thus, the cell line dependence of the Young’s modulus is consistent with observations made in cell lines of different (non-)tumor entities. If the composite parameter “generalized stiffness” is taken as a measure of cellular elasticity, an increase in both cell lines after irradiation was observed. The data are in agreement with the general notation of an association of an increased stiffness with a less aggressive phenotype, as observed in this study [12,13,14,15,48,52,53,54,55,56,57,58,59,60,61,62,63,64]. 

### 3.3. Effects of 2 Gy Single-Dose Radiation on Tumor Invasiveness, the Composite Parameter Stiffness, and the Actin Cytoskeleton

When evaluating the invasiveness of both cell lines, a decrease after single-dose irradiation of 2 Gy was observed for both time points. This is in agreement with studies from other groups, showing a decrease in invasiveness after conventional photon beam irradiation [65,66]. These results are also supported by the reduced growth rate after irradiation reported by other groups and, in the case of the U87 cells, by the reduced proliferation index found here [33]. Therefore, in the beginning or shortly after the start of the tumor invasion study, the irradiated cells had a reduced growth rate and thus the resulting tumor mass decreased. Other studies implied that non-lethal radiation doses may lead to a more aggressive phenotype in U87 glioblastoma cells [67,68,69]. Two of the main differences between these studies and the results obtained here are the time frame and the model system. Two of these studies used a transwell assay with an observation time of 3 or 24 h, thus allowing a far lower invasion time [67]. Additionally, the transwell assay may be flawed by single cell effects, such as an increase in cell motility, as was detected here for U87 cells or changes in cellular elasticity or contractility. Furthermore, the transwell assay does not provide a physiological milieu that is comparable to the central nervous system, raising further questions about comparability with the model used in this study. In contrast to that, the publication of Shankar et al. used a rat in vivo model with a single irradiation dose of 50 Gy and evaluated the tumor size seven weeks after irradiation [68]. Thus the time between irradiation and evaluation of the invasiveness is much higher, allowing the surviving cell fraction to adapt and potentially change its growth characteristics. Additionally, in standard therapy a maximum dose of 60 Gy is applied in fractions of 2 Gy over six weeks, resulting in an average of approximately 1.5 days between successive radiation events [70]. The approach used here mimics the clinical conditions better in the sense of fractionation and is hence hard to compare with the model of Shankar et al. [68].

We found in our study that the generated composite parameter stiffness increased for both cell lines after irradiation. This parameter also correlates negatively with the measured invasiveness and agrees with the negative correlation of the composite parameter stiffness with the invasiveness found before by our lab [24]. This relation is further supported by the data obtained by other research groups, observing a negative correlation between cell stiffness and tumor aggressiveness in various tumor types, like mammary carcinoma, cervix carcinoma, prostate carcinoma, melanoma, etc. [12,13,14,15,48,52,53,54,55,56,57,58,59,60,61,62,63,64]. As the cell stiffness is strongly related to the cytoskeleton and especially actin structures, we evaluated the structure of the actin cytoskeleton. Thereby we could observe that the irradiation alters the quality of actin structures in a cell line-dependent manner, and only in U87 cells the structure density was increased. This shows that the effects on cell speed and stiffness are possibly mediated via changes in the amount, length, and/or quality of actin fibers. However, the exact mechanisms behind these structural changes remain unclear, as the structure analysis is based on anisotropy and is thus influenced by the size, amount, and brightness of structures. These properties can either be influenced by the ratio of G-actin to F-actin or via the polymerization dynamics of the observed actin fibers or the activity of cross-linkers [71]. The observed change in elastic modulus and actin organization is in agreement with previously reported results from different tumor types, pointing out that for small cellular deformations, as has been observed here, actin is the main contributor to cellular elasticity [14,15,55]. Combining the obtained information about the influence of the actin cytoskeleton with the negative correlation of the composite parameter stiffness with the invasiveness, we could demonstrate that irradiation may mediate a portion of its effects on tumor invasion directly via the cytoskeleton and alterations in its organization.

In summary, we demonstrate that non-lethal irradiation can lead to changes in the cytoarchitecture of two glioblastoma cell lines, with a reduced generalized cell stiffness and invasiveness for 3–4 days after cell application. We furthermore extend the previously established negative correlation between the invasiveness and the composite parameter stiffness. This approach possibly allows a qualitative prediction of the effectiveness of glioblastoma treatments by a simple and quick measurement of the biomechanical properties of single cells. However, as glioblastoma are highly heterogeneous, it is necessary to validate the proposed hypothesis in further cell lines and systems that reflect in vivo conditions better, like primary glioblastoma cells and glioma stem cells [72,73]. 

## 4. Materials and Methods

### 4.1. Cell Culture and Irradiation

U87 and LN229 cells were obtained from the American Type Culture Collection (ATCC, Manassas, VA, USA; U87: ATCC^®^ HTB-14^TM^; LN229: ATCC^®^ CRL-2611^TM^). Both cell lines were cultured as described elsewhere [24]. 24 h prior to the start of experiments, the culture medium was changed and the cells were irradiated with 2 Gy, with a dose rate of 2 Gy/min using 6 MV photons (Siemens, Erlangen, Germany, Siemens ONCOR). 

### 4.2. Immunofluorescence and Immunohistochemical Staining

24 h after the irradiation, 50,000 cells were placed on glass cover slips coated with poly-l-lysin (Carl Roth, Karlsruhe, Germany) and incubated for another 24 h until fixation with 4% paraformaldehyde for 10 min. 

For one group, propidium iodide (PI, 5 µg/mL, Merck Millipore, Billerica, MA, USA, 537059) was added to the culture medium 2 h before fixation. For phalloidin-488 staining, another group of cells was incubated with normal goat serum (Sigma Aldrich, Saint Louis, MI, USA, G9023) in PBS/Triton for 30 min, then for 5 min in 0.1% PBS/Triton solution, washed with PBS, and blocked with 1% bovine serum albumin. An incubation step with phalloidin-488 (2.5 µL/100 µL BSA solution, Thermo Fisher Scientific, Waltham, MA, USA, A12379) was performed for 20 min. For the visualization of the nucleus, Sytox Green (1:10,000, Thermo Fisher Scientific, S7020) or 4′,6-Diamin-2-phenylindol (DAPI, 1:10,000, Sigma Aldrich, D9542) was used. The stained cells were finally washed with both PBS and distilled water and covered with DAKO mounting medium (DAKO, Santa Clara, CA, USA, S302380).

The number of PI-positive, dead cells was counted and divided by the number of Sytox Green positive cells. At least 100 Sytox Green positive cells per coverslip were counted. 

The assessment of the proliferation index was performed as described before [74] with an antibody against Ki67 (1:200, rabbit, DCS-Innovative Diagnostik-Systeme, Hamburg, Germany, EPR3611). A subsequent staining with hematoxylin was performed and the slides were covered with Entellan (Merck, Kenilworth, NJ, USA, 107961). All images were obtained at 400× magnification with an Axioplan microscope (Zeiss, Oberkochen, Germany, Axioplan). Five different regions per cover slip were analyzed. The number of Ki67 and hematoxylin positive cells was counted and the proliferation index calculated.

### 4.3. Western Blotting

The Western blot analysis was performed as described before [24]. The cells were collected in 75 µL of sample buffer. Ten micrograms of the sample were loaded on the electrophoresis gel. Anti-GAPDH (37 kDa) antibody (Cell Signaling, Cambridge, UK, 14C10, 1:1000) was used as housekeeping protein and PCNA (Santa Cruz, Dallas, TX, USA, 1:1000, PC10, 36 kDa) for the assessment of proliferation. The imaging and evaluation of blots was performed using the Fusion FX7 (PeqLab, Erlangen, Germany).

### 4.4. Time Lapse Microscopy

For live cell imaging experiments cells were irradiated, kept in culture for 24 h, and seeded (1000 cells) in a six-well plate 24 h before the start of the experiments. Images were acquired every 5 min using a microscope (Leica, Wetzlar, Germany, Leica DMi 8) with temperature (37 °C) and CO_2_ regulation (5% (*v*/*v*)). The experiments were conducted as described previously and for the evaluation the parameters cell area, mean squared displacement, directionality, persistence time, persistence speed, and mean speed were measured (see Supplementary Materials) [24].

### 4.5. Atomic Force Microscopy

For measuring the Young’s modulus, an atomic force microscope (AFM; Bruker, Billerica, MA, USA, Bioscope Catalyst) was used. Cells that were allowed to adhere to a petri dish for 15 min were used for the experiments. Single cells were measured with a tip-less cantilever (Arrow-TL2, Nanoworld, Hong Kong, China) using a force of 3 nN. The Young’s modulus was calculated with the Hertz model, while the Derjarguin–Muller–Topolov model was used for estimating the normalized adhesion energy [75]. Additionally, the parameters indentation, jump energy, total adhesion energy, minimal force, jump force, slope of approach curve, cell radius, and jump number were obtained (Tables S1 and S2), as explained elsewhere [24].

### 4.6. Organotypic Hippocampal Slice Cultures (OHSC) and Tumor Invasion

All experiments involving animal material were performed in accordance with the directive 2010/63/EU of the European Parliament and the Council of the European Union (22.09.2010).

OHSC were prepared from five-day-old C57 Black6/J mice as reported earlier and kept at 35 °C in a fully humidified atmosphere with 5% (*v*/*v*) CO_2_ [76]. The culture medium was changed every other day. Thirteen days after preparation of OHSC, tumor cells were irradiated or left untreated. 24 h later, the experiments were started. Irradiated or non-irradiated cells (50,000) were labeled with carboxyfluorescin diacetate (CFDA; Gibco, Waltham, MA, USA, 12883), placed onto the slice cultures, and allowed to invade for a further three or four days. Afterwards, the co-culture was fixed using 4% PFA. The cytoarchitecture of the slice was labeled with PI, as previously reported [24,77,78,79].

### 4.7. Confocal Laser Scanning Microscopy (CLSM)

Images of the fixed OHSC were acquired with a 10× objective and phalloidin-stained cells with a 63× objective using a confocal laser scanning microscope. The following excitation wavelengths were used: 405 nm for DAPI, 488 nm for CFDA and phalloidin, and 543 nm for PI. Emission was detected in the range of Δλ = 400–480 nm (DAPI), Δλ = 510–550 nm (CFDA), Δλ = 500–650 nm (phalloidin), and Δλ = 610–720 nm (PI).

For tumor invasion measurement, images were obtained as z-stacks with a step width of 2 µm. The resulting images of OHSC were evaluated using the maximal intensity projection with a subsequent thresholding to calculate the area of the OHSC covered by tumor cells. 

For evaluation of cytoskeletal alterations, we used an approach described elsewhere that is based on the image coherency [80]. This approach assumes that the overall structure can be understood as the sum over all local structures of actin fibers inside the cell. Thereby two quantities are obtained: the structure density and its quality. The quality can be understood as the contrast of fibers and is thus coupled to fiber thickness. The images were analyzed using a self-written MATLAB (The MathWorks, Natick, MA, USA) script.

### 4.8. Network Analytical Approach

Data obtained by AFM and live cell microscopy were used to quantify the relationship between the cell-specific parameters. Therefore, a network analytical approach was used, as reported previously [81]. Briefly, communities *C* were generated in such a way that the obtained network deviates most from a randomly formed network with the same number of nodes and edges [82]. To reduce the number of parameters for the later analysis composite parameters were introduced [81]. The composite parameter *M^C^* is the “sum” of all parameters in a community *C* normalized regarding their mean and standard deviation:

(1) $$M^{C}=\frac{1}{N_{C}}\underset{K\in C}{\sum }\frac{\left[m_{K}\right]-\left(m_{K}\right)}{\sigma \left(m_{K}\right)}$$

where [*m_K_*] are single measurements of parameter *K*, and *N_C_* is the number of parameters in community *C*.

### 4.9. Statistics

Statistical analysis was performed using the two-sided Mann–Whitney–Wilcoxon test or *t*-test and significance was chosen for *p* < 0.05. All *p* values refer to the respective controls of the same parameter of the same cell line.

## 5. Conclusions

We could demonstrate that non-lethal irradiation can lead to alterations in the cytoarchitecture of glioblastoma cells, leading to a reduced stiffness that is associated with a decrease in invasiveness. The presented approach may possibly allow a qualitative prediction of the effectiveness of glioblastoma treatments by measuring biomechanical properties of single cells in the future. 

## Supplementary Materials

Supplementary materials can be found at www.mdpi.com/1422-0067/18/9/2001/s1.

## Abbreviations

AFM	Atomic force microscope
CFDA	Fluorophores carboxyfluorescin diacetate
CTL	Control
EGFR	Epidermal growth factor receptor
FBS	Fetal bovine serum
GBM	Glioblastoma multiforme
OHSC	Organotypic hippocampal slice culture
PBS	Phosphate buffered saline
PCNA	Proliferating cell nuclear antigen
PI	Propidium iodide
P/S	Penicillin/streptomycin
sem	Standard error of the mean
