# Supplementary material for: The Impact of Non-Lethal Single-Dose Radiation on Tumor Invasion and Cytoskeletal Properties
Source: Int J Mol Sci. 2017 Sep 18;18(9):2001. doi: 10.3390/ijms18092001 (PMC5618650; doi:10.3390/ijms18092001)
Supplement: Supplementary file 1 [file ijms-18-02001-s001.zip › suppl..pdf]

Supplemental Information

Materials and Methods:

List of parameters for the live cell measurements:

Table S1. explains the parameters determined from the live cell imaging.

Table S1. Measurement parameters of the live cell imaging.

#	Parameter Name	Description
1	Cell Area	Average area of a cell during the measurement (24 h).
2	Mean Squared Displacement	Mean squared displacement after 24 h.
3	Directionality	Quotient of the distance between the start and endpoint of the cell's movement and the incremental distance traveled.
4	Persistence Time	Parameter of the Fürth formula: $< x^2 > = \frac{2v_p^2}{t_p^2}(t_p\, t + e^{-t_p t} - 1)$, with persistence time t_p, persistence speed v_p and time t. The persistence time is the time a cell's movement is roughly oriented in one direction.
5	Persistence Speed	Parameter v_p of the Fürth formula.
6	Mean Speed	Average speed of the cell during the measurement (24 h).

List of AFM parameters:

The parameters extracted from the atomic force microscope measurements are listed and briefly explained in Table S2.

Table S2. Measurement parameters of the AFM measurements.

#	Parameter Name	Description
1	Indentation	Physical deformation
2	Normalized Adhesion Energy	Adhesion energy per unit area calculated using the Derjarguin–Muller–Toporov (DMT) model.
3	Jump Energy	Energy of a discrete jump in the retract curve (integral between the start and end).
4	Total Adhesion Energy	Energy of adhesion determined for the first and second zero-passing of the retract curve.
5	Minimal or Rupture Force	Highest negative value of the retract curve.
6	Jump Force	Difference in force between the start and end of a discrete jump in the retract curve.
7	Slope of Approach Curve	Slope of the first 240 nm of the approach curve.
8	Cell Radius	Radius of the cell before the AFM measurement.
9	Young's Modulus	Elastic modulus determined with the Hertz model.
10	Jump Number	Number of discrete jumps in the retract curve.

Results:

Network analytical results:

The parameter groupings for the AFM and live cell measurements are summarized in Figure S2. If the mutual information between two parameters was larger than 0, an edge was created between vertices.

In the case of live cell imaging data, we observed a share of information between the persistence speed and mean speed only (Figure S1A).

In contrast to that, three parameter clusters were found for the AFM measurements (Figure S1B). The indendation depth was clustered with the Young's modulus. All adhesive properties (total adhesion energy, minimal force, and normalized adhesion energy) formed a further cluster. The last cluster was generated by two parameters describing discrete jumps in the retract curve: energy and force of these jumps.

Representative Videos for Live Cell Imaging Experiments:

Attached are image series of live cell experiments depicting the typical movement patterns of LN229 and U87 cells with and without irradiation of 2 Gy.

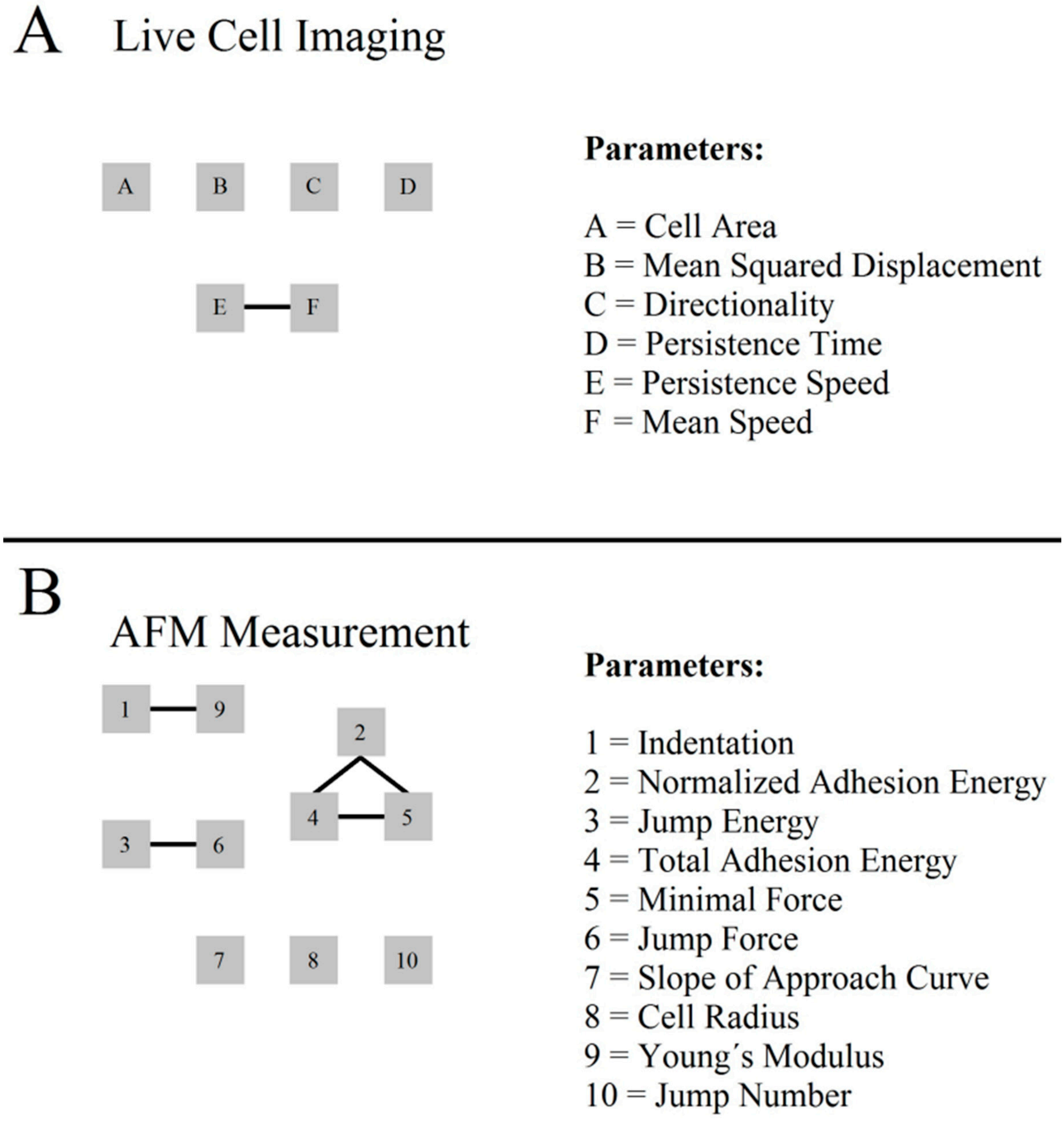

Figure S1: Scheme of the network analysis results. The squares depict vertices and the edges are shown as lines connecting the vertices. Numbers and letters represent the listed parameters. Top: List of parameters from the time lapse image experiments. Bottom: List of parameters gained from analyzing atomic force microscopy measurements.
